# Association between thyroid function and diabetic nephropathy in euthyroid subjects with type 2 diabetes mellitus: a cross-sectional study in China

**DOI:** 10.18632/oncotarget.26265

**Published:** 2019-01-04

**Authors:** Jian Wang, Huiqin Li, Mingjuan Tan, Gu Gao, Ying Zhang, Bo Ding, Xiaofei Su, Xiaocen Kong, Jianhua Ma

**Affiliations:** ^1^ Department of Endocrinology, Nanjing First Hospital, Nanjing Medical University, Nanjing 210012, China; ^2^ Department of Clinical Laboratory, Nanjing First Hospital, Nanjing Medical University, Nanjing 210012, China

**Keywords:** thyroid hormones, free triiodothyronine, free thyroxine, thyroid-stimulating hormone, type 2 diabetes mellitus

## Abstract

Previous studies have suggested that even in euthyroid subjects, thyroid function may affect the risk factors of diabetic nephropathy (DN). Thus, we investigated the association between thyroid parameters and DN in euthyroid subjects with type 2 diabetes mellitus (T2DM). This was a cross-sectional study of 1,071 euthyroid subjects with T2DM (mean age of 61.90 ± 12.74 years; 622 men). Clinical factors, including levels of free triiodothyronine (FT3), free thyroxine (FT4), thyroid-stimulating hormone (TSH), thyroid autoantibodies, albumin excretion rate were measured. DN was present in 400 (37.35%) individuals. Patients with DN exhibited higher serum TSH and lower serum FT3 and FT4 levels than those without DN (*P<*0.05). After adjusting traditional risk factors of DN, the levels of both FT3 (per-SD increase, odds ratio [OR] 0.606 [95% confidence interval (CI), 0.481–0.762], *P*<0.001) and FT4 (per-SD increase, OR 0.944 [0.894–0.998], *P* = 0.040) were inversely correlated with DN. Meanwhile, we found that serum TSH levels were positively correlated with DN (per-SD increase, OR1.179 [1.033–1.346], *P* = 0.015). Low-to-normal thyroid hormones (THs) were also associated with the presence of macroalbuminuria. In conclusion, the relatively low levels of THs were significantly associated with DN in euthyroid subjects with T2DM.

## INTRODUCTION

The prevalence of diabetes continues to increase significantly worldwide, especially in developing countries [1, 2]. This leads to an increase in the development of diabetic nephropathy (DN) [3]. As one of the main microvascular complications of diabetes, DN is the leading cause of end-stage renal disease (ESRD), which contributes to a significant increase in morbidity and mortality in diabetic patients [3]. One of the characteristics of DN is the development of albuminuria, which followed by a progressive decline in kidney function. When persistent albuminuria occurs, conventional therapies (such as smoking cessation, improved glycemic and blood pressure control) may not be able to fully terminate the progression of DN [4]. Therefore, it is essential to identify the risk factors of DN and monitor its development or progression, which will improve the treatment efficacy and reduce individual and socioeconomic burdens of the disease.

The impact of thyroid hormones (THs) on DN has recently aroused the interests. THs play important roles in the regulation of renal development, kidney hemodynamics, glomerular filtration rate (GFR) and sodium and water homeostasis [5]. Hyperthyroidism is accompanied by increased renal blood flow and GFR [6]. Overt and subclinical hypothyroidism (SCH) may lead to renal dysfunction via decreased GFR as well as to alterations in heart and vascular function and derangements in the renin-angiotensin system [6–8]. Several clinical studies [9, 10] have reported that SCH is also associated with the development of both DN and microalbuminuria. In addition, some prospective studies [11, 12] have shown that levothyroxine (LT4) treatment can significantly decrease the urinary albumin-to-creatinine ratio (UAER) and has protective effects on the kidneys in patients with both DN and SCH.

Several recent studies [13, 14] have reported that low-to-normal THs are associated with the risk factors of DN, such as hypertension, plasma lipid levels and endothelial function. Furthermore, some longitudinal studies [15, 16] have demonstrated the correlation between low-to-normal THs and the incidence of macrovascular diseases. Some research results suggested a lower TSH target (e.g., 0.40 to 2.50 mIU/L) for SCH patients [17]. However, it is still unknown whether there is an association between relatively low levels of THs and DN among euthyroid subjects with type 2 diabetes mellitus (T2DM). In this study, we assess the potential associations between thyroid parameters and DN in euthyroid subjects with T2DM.

## RESULTS

### Clinical and biochemical characteristics of the study subjects

For the total 1,071 participants, 671 patients (62.65%) had a normal AER, 312 patients (29.13%) had microalbuminuria, and 88 patients (8.22%) had macroalbuminuria. The clinical and laboratory characteristics of the study patients areshown in Table 1. Compared to patients with normoalbuminuria, patients with either micro- or macroalbuminuria were older, tended to be current smokers, and had hypertension, hyperlipidemia, a longer diabetes duration, higher BMI, higher SBP and DBP, higher TG, BUN, and SCr levels, and a lower eGFR. In addition, serum FT3, FT4, and TSH levels were significantly different among the three groups (*P*< 0.001, *P*< 0.001, and *P* = 0.002, respectively). Patients with micro- or macroalbuminuria were more likely to have higher serum TSH and lower serum FT3 and FT4 levels. All other characteristics did not significantly differ between groups.

**Table 1:** Clinical and biochemical characteristics of the study patients

Characteristics	Total (n=1071)	Normoalbuminuria (n=671)	Mircoalbuminuria (n=312)	Macroalbuminuria (n=88)	*P*-Value
Age (years)	61.90 ± 12.74	60.69 ± 12.85	63.61 ± 12.36^*^	65.02 ± 12.08^*^	<0.001
Male, n (%)	622 (58.08%)	372 (55.44%)	195 (62.5%)^*^	55 (62.5%)	0.077
Current smokers (*n* (%))	259 (24.18%)	145 (21.61%)	87 (27.88%)^*^	27 (30.68%)^*^	0.034
Current alcohol (*n* (%))	120 (11.20%)	68 (10.13%)	42 (13.46%)	10 (11.36%)	0.305
Hypertension (*n* (%))	606 (56.58%)	335 (49.92%)	205 (65.70%)^*^	66 (75%)^*^	<0.001
Hyperlipoidemia (*n* (%))	623 (58.17%)	367 (54.69%)	198 (63.46%)^*^	58 (65.91%)^*^	<0.001
Family history of T2DM (n (%))	293 (27.36%)	178 (26.53%)	91 (29.16%)	24 (27.27%)	0.688
Diabetes duration (years)	6.00 (1.00, 10.00)	5.00 (0.50, 10.00)	7.00 (3.00, 12.00)^*^	10.00 (4.00, 15.75)^*#^	<0.001
BMI (kg/m^2^)	24.74 ±3.30	24.49 ± 3.13	25.03 ± 3.35^*^	25.60 ± 4.10^*^	0.002
SBP (mmHg)	135.10 ± 17.03	132.43 ± 14.53	137.66 ± 16.33^*^	146.39 ±27.95^*#^	<0.001
DBP (mmHg)	81.74 ± 9.85	80.87 ± 8.88	82.40 ± 10.10^*^	86.01 ± 14.01^*#^	<0.001
HbA1c (%)	9.06 ± 2.16	9.05 ± 2.19	9.13 ± 2.14	8.97 ± 2.04	0.795
ALT (u/L)	21.00 (15.00, 31.00)	21.00 (16.00, 31.00)	22.00 (15.00, 32.00)	21.00 (15.25, 28.00)	0.748
AST (u/L)	20.00 (16.00, 26.00)	20.00 (16.00, 26.00)	20.00 (15.00, 26.00)	22.00 (17.00, 26.75)	0.963
TC (mmol/L)	5.05 (4.43, 5.90)	5.03 (4.45, 5.78)	5.04 (4.27, 6.07)	5.30 (4.60, 6.14)^*^	0.103
TG (mmol/L)	1.43 (1.00, 2.25)	1.39 (0.99, 2.15)	1.48 (1.02, 2.28)	1.61 (1.08, 2.57)^*#^	0.012
HDL-C (mmol/L)	1.27 (1.02, 1.52)	1.27 (1.03, 1.52)	1.27 (1.02, 1.52)	1.26 (0.92, 1.55)	0.949
LDL-C (mmol/L)	2.71 ±0.81	2.71 ±0.79	2.69 ± 0.85	2.77 ±0.81	0.705
FT3 (pmol/L)	4.37 ±0.63	4.44 ±0.60	4.32 ±0.65^*^	4.02 ±0.65^*#^	<0.001
FT4 (pmol/L)	15.33 ± 2.49	15.51 ± 2.49	15.20 ± 2.46	14.42 ± 2.39^*#^	<0.001
TSH (*μ*IU/mL)	1.63 (1.13, 2.37)	1.58 (1.12, 2.32)	1.71 (1.11, 2.53)	1.85 (1.34, 2.96)^*#^	0.002
BUN (*μ*mol/L)	6.41 ± 2.69	5.97 ± 2.08	6.72 ± 3.05^*^	8.69 ± 3.86^*#^	<0.001
SCr (*μ*mol/L)	71.90 (59.00, 90.00)	67.00 (57.00, 83.00)	76.00 (62.00, 90.75)^*^	110.50 (80.85, 145.75) ^*#^	<0.001
eGFR (ml/min/1.73m^2^)	92.32 (72.96, 114.70)	97.26 (72.96, 114.70)	67.36 (33.88, 86.96)^*^	57.23 (41.03, 81.92)^*#^	<0.001
AER (mg/24h)	19.00 (9.77, 54.00)	12.03 (7.70, 18.00)	61.56 (40.47, 115.68)^*^	466.55 (301.00, 761.25)^*#^	<0.001

Abbreviations: T2DM, type 2 diabetes mellitus; BMI, body mass index; SBP, systolic blood pressure; DBP, diastolic blood pressure; HbA1c, glycated hemoglobin; ALT, alanine aminotransferase; AST, aspertateaminotransferase; TC, total cholesterol; TG, triglyceride; HDL-C, high-density lipoproteincholesterol; LDL-C, low-density lipoproteincholesterol; FT3, free triiodothyronine; FT4, free thyroxine; TSH, thyroid stimulating hormone; BUN, urea nitrogen; SCr, serum creatinine; eGFR, estimated glomerular filtration rate; AER, albumin excretion rate.

Diabetes duration, ALT, AST, TC, TG, HDL-C, TSH, SCr, eGFR and AER were log10-transformed because ofnon-normal distribution.

^*^significantly different (P<0.05) from the patients with nomoalbuminuria; #significantly different (P<0.05) from the patients with mircoalbuminuria.

### Association of thyroid function with DN

The univariate logistic regression analyses showed that traditional risk factors for DN, including age (odds ratio [OR] 1.021, [95% confidence interval {CI}, 1.010 – 1.031], *P* < 0.001), somking (OR 1.446, [95% CI, 1.088 – 1.922], *P* = 0.011), hyperlipidemia (OR 1.463, [95% CI, 1.134 – 1.887], *P* = 0.003), duration of diabetes (OR 1.057, [95% CI, 1.037 – 1.078], *P*< 0.001), BMI (OR 1.063, [95% CI, 1.024 –1.104], *P* = 0.001), SBP (OR 1.026, [95% CI, 1.018 – 1.034], *P*< 0.001), and eGFR (OR 0.986, [95% CI, 0.982 – 0.990], *P* < 0.001), were significantly associated with DR.

Table 2 shows the associations of serum FT3, FT4, and TSH levels with DN. After adjusting for all covariables in model 2, both FT3 (OR, 0.606 [95% CI, 0.481–0.762], *P*<0.001) and FT4 (OR 0.944, [95% CI, 0.894–0.998], *P* = 0.040) levels were inversely associated with DN. Patients with the highest tertile of serum FT3 (FT4) levels had a 0.452- (0.666-) times probabilty of developing DN compared to those with the lowest tertile of serum FT3 (FT4) levels (*P*_trend_ = 0.003 and *P*_trend_ = 0.052, respectively). Meanwhile, we found that serum TSH levels were positively associated with DN risk (OR1.179 [95% CI, 1.033–1.346], *P* = 0.015; highest versus lowest tertile, OR 1.392 [95% CI, 1.004–1.932]; *P*_trend_ = 0.022).

**Table 2:** Association of thyroid status with diabetic nephropathy

Thyroid status	Diabetic nephropathy			
	Model 1		Model 2	
	OR (95%CI)	*P*-Value	OR (95%CI)	*P*-Value
FT3 (pmol/L)				
1^st^tertile, ≤ 4.10	1.000 (referent)	<0.001^*^	1.000(referent)	<0.001^*^
2^nd^tertile, 4.10 – 4.65	0.519 (0.380 – 0.708)	<0.001	0.515 (0.370 – 0.719)	<0.001
3^rd^tertile, > 4.65	0.488 (0.353 – 0.675)	<0.001	0.452 (0.318 – 0.642)	<0.001
Per SD-increase	0.637 (0.516 – 0.788)	<0.001	0.606 (0.481 – 0.762)	<0.001
FT4 (pmol/L)				
1^st^tertile, ≤ 14.26	1.000(referent)	0.026^*^	1.000(referent)	0.052^*^
2^nd^tertile, 14.26 – 16.30	0.883 (0.652 – 1.197)	0.423	0.878 (0.635 – 0.1.215)	0.432
3^rd^tertile, > 16.30	0.655 (0.479 – 0.896)	0.008	0.666 (0.477 – 0.930)	0.017
Per SD-increase	0.938 (0.891 – 0.987)	0.014	0.944 (0.894 – 0.998)	0.040
TSH (*μ*IU/mL)				
1^st^tertile, ≤ 1.30	1.000(referent)	0.016^*^	1.000(referent)	0.022^*^
2^nd^tertile, 1.30 – 2.07	0.930 (0.680 – 1.271)	0.648	0.892 (0.642 – 1.242)	0.499
3^rd^tertile, > 2.07	1.420 (1.043 – 1.934)	0.026	1.392 (1.004 – 1.932)	0.047
Per SD-increase	1.176 (1.040 – 1.331)	0.010	1.179 (1.033 – 1.346)	0.015

Each risk factor is in separate models. Model 1: adjusted for age and sex. Model 2:model 1 plus adjusted for smoking status (yes/no), hyperlipoidemia (yes/no), duration of diabetes, BMI, SBP, HbA1c, eGFR. Age, duration of diabetes, BMI, SBP, HbA1c, eGFRwere treated as continuous variables.

^*^P_trend_.

**Figure 1:**
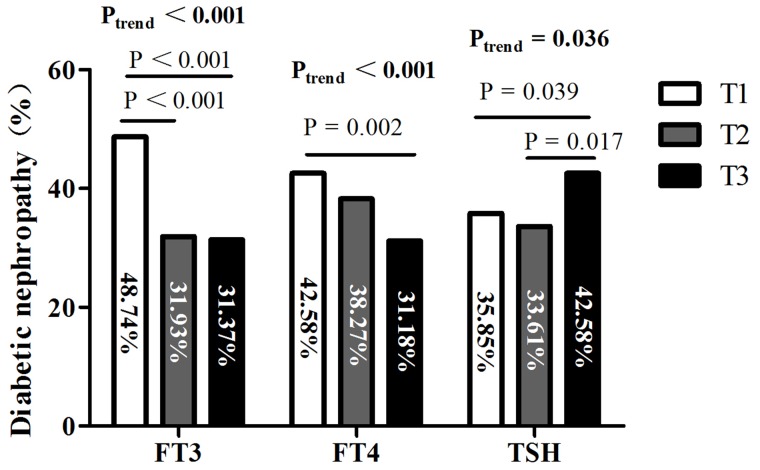
Prevalence of diabetic nephropathy (DN) among tertiles based on FT3, FT4, and TSH levels Abbreviations: FT3, free triiodothyronine; FT4, free thyroxine; TSH, thyroid stimulating hormone; T1, tertile 1; T2, tertile 2; T3, tertile 3.

After that, we evaluated differences in the prevalence of DN among the FT3, FT4, and TSH tertile groups (Figure 1). The prevalence of DN was 48.74% of patients in the lowest tertile of serumFT3 levels, which was much higher than those in the other two groups (*P*_trend_< 0.001). The prevalence of DN also showed a significantly decreasing trend across the three tertiles according to FT4 levels (42.58%, 38.27%, and 31.18%, *P*_trend_=0.006). However, the prevalence of DN increased across the three tertiles according to TSH levels (35.85%, 33.61%, and 42.58%, *P*_trend_=0.036).

### Association between thyroid function and the presence of macroalbuminuria

Table 3 shows the associations of serum FT3, FT4, and TSH levels with the presence of macroalbuminuria. After adjusting for all covariables (model 2), the per-SD increasein TSH was associated with an increased probability of macroalbuminuria (OR 1.376, [95% CI, 0.894–0.998], *P* = 0.007). Likewise, decreasing levels ofboth FT3 (OR 0.413 [95% CI, 0.270–0.630], *P*<0.001; highest versus lowest tertile, OR 0.336 [95% CI, 0.167–0.674]; *P*_trend_ = 0.008) and FT4 (OR0.856 [95% CI, 0.768–0.953], *P* = 0.005; highest versus lowest tertile, OR0.296 [95% CI, 0.152–0.576]; *P*_trend_ = 0.001) were associatedwith an increased probability of macroalbuminuria.

**Table 3:** Association of thyroid status with the presence of macroalbuminuria

Thyroid status	The presence of macroalbuminuria			
	Model 1		Model 2	
	OR (95%CI)	*P*-Value	OR (95%CI)	*P*-Value
FT3 (pmol/L)				
1^st^tertile, ≤ 4.10	1.000 (referent)	<0.001^*^	1.000(referent)	0.008^*^
2^nd^tertile, 4.10 – 4.65	0.487 (0.288 – 0.821)	0.007	0.618 (0.343 – 1.114)	0.110
3^rd^tertile, > 4.65	0.307 (0.166 – 0.566)	<0.001	0.336 (0.167 – 0.674)	0.002
Per SD-increase	0.393 (0.275 – 0.563)	<0.001	0.413 (0.270 – 0.630)	<0.001
FT4 (pmol/L)				
1^st^tertile, ≤ 14.26	1.000(referent)	0.001^*^	1.000(referent)	0.001^*^
2^nd^tertile, 14.26 – 16.30	0.577 (0.349 – 0.954)	0.032	0.529 (0.297 – 0.944)	0.031
3^rd^tertile, > 16.30	0.318 (0.173 – 0.584)	<0.001	0.296 (0.152 – 0.576)	<0.001
Per SD-increase	0.862 (0.786 – 0.945)	0.001	0.856 (0.768 – 0.953)	0.005
TSH (*μ*IU/mL)				
1^st^tertile, ≤ 1.30	1.000(referent)	0.046^*^	1.000(referent)	0.131^*^
2^nd^tertile, 1.30 – 2.07	1.641 (0.914 – 2.945)	0.097	1.673 (0.874 – 3.202)	0.120
3^rd^tertile, > 2.07	2.059 (1.162 – 3.651)	0.013	1.882 (0.999 – 3.546)	0.047
Per SD-increase	1.401 (1.155 – 1.699)	0.001	1.376 (1.093 – 1.732)	0.007

Each risk factor is in separate models. Model 1: adjusted for age and sex. Model 2:model 1 plus adjusted for smoking status (yes/no), hyperlipoidemia (yes/no), duration of diabetes, BMI, SBP, HbA1c, eGFR. Age, duration of diabetes, BMI, SBP, HbA1c, eGFRwere treated as continuous variables.

^*^P_trend_.

**Figure 2:**
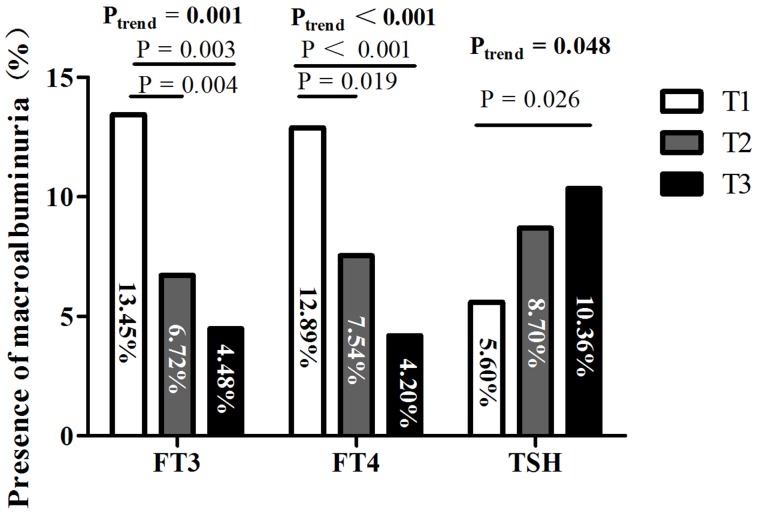
Presence of macroalbuminuria among tertiles based on FT3, FT4, and TSH levels Abbreviations: FT3, free triiodothyronine; FT4, free thyroxine; TSH, thyroid stimulating hormone; T1, tertile 1; T2, tertile 2; T3, tertile 3.

The presence of macroalbuminuria decreased across the three tertiles according to FT3 (*P*_trend_= 0.001) and FT4 (*P*_trend_< 0.001) levels and increased across the three tertiles according to TSH (*P*_trend_= 0.048) levels (Figure 2).

## DISCUSSION

In this hospital-based, cross-sectional study, we evaluated the associations between thyroid parameters and DN in euthyroid subjects with T2DM. Our results showed that subjects with DN had lower FT3 and FT4 levels and higher TSH levels than those with normoalbuminuria. After adjusting for potential risk factors of DN (age, sex, BMI, smoking status, the duration of T2DM, SBP, hyperlipidemia, HbA1c level, and eGFR), low-to-normal FT3 and FT4 levels, and high-to-normal TSH levels, were significantly associated with DN. Low-to-normal THs were also associated with the presence of macroalbuminuria, which indicated a severe stage of DN. These findings indicate that low-to-normal TH levels may be potential risk factors for DN development.

Clinical trials have consistently shown that both hypertension and poor glycemic control are associated with DN [18]. In the United Kingdom Prospective Study (UKPDS), the incidence of DN was significantly associated with SBP [19]. Each 10-mm Hg decrease in SBP was associated with a 13% reduction in microvascular complications. Comprehensive prospective data from another study [20] also showed that glycemic control (an HbA1c level < 7%) was associated with reduced microvascular injury. In the UKPDS, the intensively treated group showed a 30% risk reduction for the development of microalbuminuria [21]. Consistent with these results, we found that patients with DN had a higher SBP than patients without DN. Although there was no significant difference in HbA1c levels between patients with and without DN, we further compared the incidence of DN between patients with poor glycemic control (HbA1c ≥ 7%) and those with optimal glycemic control (HbA1c < 7%). It was found that the incidence of DN in patients with an HbA1c greater than or equal to 7% was higher than those with an HbA1c level less than 7% (39.7% vs. 25.6%, *P*< 0.001). In addition, previous studies [22, 23] have reported that age, smoking, obesity, hyperlipidemia, and prolonged duration of diabetes are also risk factors for DN, which are consistent with our results.

In addition to the traditional risk factors for DN, other studies [24, 25] have indicated that thyroid function is closely related to the occurrence of DN. Several studies [25] have reported an increased prevalence of DN in patients with diabetes also suffered from hypothyroidism or SCH. Furthermore, LT4 replacement therapy might reduce the risk of DN, such as reducing blood lipid and uric acid levels, improving renal ischemia by increasing GFR, and delaying the progression of kidney disease in such patients [11, 12]. An additional study [26] reported a high prevalence of nonautoimmune, primary hypothyroidism in diabetic patients with advanced DN compared to those with nondiabetic kidney dysfunction, suggests that such hypothyroidism might be an important contributing factor to the early development of edema. In our study, we observed that low-to-normal TH levels were significantly associated with higher AER and lower eGFR, even in euthyroid diabetic subjects.

Two hypotheses are suggested to explain the association between low-to-normal TH levels and DN. The first hypothesis is that low-to-normal TH levels can cause DN via both direct and indirect effects. Firstly, low TH levels could lead to DN indirectly by causing hypertension and hyperlipidemia, conditions that hypothyroid and SCH patients often exhibit. An11-yearfollow-up study [13] showed that high TSH levels within the reference range were associated with future high blood pressure and adverse serum lipid levels. It is generally considered that hypertension and hyperlipidemia may be the main risk factors for DN progression. Secondly, low TH levels may result in DN indirectly by affecting insulin secretion. Clinical studies [27, 28] have shown that THs are positively associated with insulin secretion in euthyroid individuals. Additional studies [29] have reported that higher insulin levels are associated with a reduced prevalence of DN in patients with T2DM. Thirdly, low TH levels may directly lead to DN by impairing vascular function. Serum FT3 levels have been shown to be associated with endothelial dysfunction as assessed by flow-mediated dilation in patients with chronic kidney disease [30]. Hypothyroid patients, even those with SCH, often experience endothelial dysfunction resulting from a reduction in nitric oxide availability, which can be reversed by LT4 supplementation [31]. Endothelial dysfunction is believed to play an important role not only in the initiation of DN but also in its progression and clinical sequelae [32]. The second hypothesis is that low-to-normal TH levels can serve as a biomarker for DN. According to previous studies [33, 34], low TH levels can occur in patients with serious diseases, and the magnitude of these alterations was found to be associated with both the severity of disease and survival outcomes. In particular, low FT3 levels have been considered as an independent predictor of mortality in patients with chronic kidney disease [35]. These observations suggest that low-to-normal TH levels can act as a biomarker for DN and indicate subsequent patient prognosis.

Macroalbuminuria, defined as an AER level greater than 300 mg/24hours, is considered as a disease state characterized by the presence of overt nephropathy and a high probability of chronic kidney disease. It is also likely to progress to ESRD in 50% of patients within 10 years and in 75% of patients by 20 years [36, 37]. Moreover, a meta-analysis [38] showed that there was a dose-response relationship between the level of albuminuria and cardiovascular disease risk. Thus, we further analyzed the association between various thyroid parameters and the presence of macroalbuminuria. After adjusting for all covariables, increasing levels of FT3 and FT4, and decreasing TSH levels, were associated with a reduced probability of macroalbuminuria. However, it is not yet known whether there is a causal relationship between thyroid status and macroalbuminuria. Further prospective studies are required to validate whether TH treatment could reduce AER in subjects with low-to-normal thyroid function and macroalbuminuria.

There are several limitations of this study. Firstly, due to its cross-sectional nature, the final causal influence of a relative TH deficit on DN in euthyroid subjects with T2DM cannot be inferred, which can only be established after a prospective analysis. Secondly, the patients were hospital-based Chinese subjects at a single center, and therefore, our results may not be applicable to all patients with T2DM. In our studies, we measured albumin excretion using 24-hour urine samples, which is considered as the gold standard for the diagnosis of DN and are more stable than a fasting urine sample. No participants had a history of thyroid disease and their thyroid autoantibody levels were negative, which negate any bias between autoimmune thyroid disease and DN.

This study on euthyroidpatients with T2DM demonstrates significant associations between low-to-normal TH levels and high TSH levels with an increased incidence of DN, especially in the presence of macroalbuminuria. Prospective studiesare guaranteed to confirm whether subjects with low-to-normal TH levelshave a higher incidence of DN.

## MATERIALS AND METHODS

### Study subjects

This was a hospital-based cross-sectional study. A total of 1,480 inpatients with T2DM, at least 18 years of age, who visited the Department of Endocrinology at Nanjing First Hospital from January 2011 to May 2013 were recruited for this study. The exclusion criteria included the following: 1) patients with overt hypothyroidism (TSH > 5.50mIU/L) or overt hyperthyroidism (TSH < 0.35mIU/L), or patients who were tested positive for thyroid autoantibodies, such as thyroid peroxidase antibody (TPOAb), thyroglobulin antibody (TGAb), or thyrotropin receptor antibody (TRAb); 2) patients with a history of chronic liver disease (liver enzyme levels more than three times higher than the upper normal limit), nondiabetic renal dysfunction, any other endocrine disorder (e.g., Addison's disease, Cushing syndrome, pituitary adenoma, orhypopituitarism), inflammatory disease, or cancer; 3) patients whose thyroid function (e.g., corticosteroids, amiodarone, carbolithium, etc.) was altered due to previously use of thyroid medications or drugs; 4) T2DM patients with acute intercurrent illness; 5) patients who are pregnantor lactating. Finally, 1,071 patients (622 men and 449 women, mean age of 61.90 ± 12.74 years) were enrolled in this study. The study was approved by the ethics committee of Nanjing First Hospital and conducted in accordance with the Declaration of Helsinki. All participants have provided the written informed consent.

### Clinical and laboratory examinations

We used a standardized questionnaire to assess patients’ clinical characteristics, including socio-behavioral information, current smoking status, family and medical histories, and medication use. Smoking was defined as a daily consumption of more than five cigarettes for at least 12 months. Height and weight were measured to determine body mass index (BMI), which was calculated as weight divided by squared height (kg/m^2^). Blood pressure was measured twice in the sitting position after a 10 minutes rest and recorded as a mean value of the two measurements. Hypertension was defined as a systolic blood pressure (SBP) greater than or equal to140 mm Hg, a diastolic blood pressure (DBP) greater than or equal to 90 mm Hg, or the current use of antihypertensive drugs. Hyperlipidemia was defined as a total cholesterol (TC) level higher than5.72mmol/L, a triglyceride (TG) level higher than 1.70mmol/L, a high-density lipoprotein cholesterol (HDL-C) level lower than 0.91 mmol/L, or the use of antihyperlipidemic agents.

Fasting (> 8 hours) blood samples were obtained to measure biochemical parameters. Levels of plasma alanine aminotransferase (ALT), aspartate aminotransferase (AST), TC, TG, HDL-C, low-density lipoprotein cholesterol (LDL-C), blood urea nitrogen (BUN), and serum creatinine (SCr) were measured by routine laboratory methods using a HITACHI7600 instrument (HITACHI, Tokyo, Japan). The estimated GFR (eGFR) was calculated using the Modification of Diet in Renal Disease equation: eGFR (mL/min/1.73 m^2^) =186 × (SCr/88.4)^−1.154^× (age)^−0.203^× (0.742 if female) [39]. HbA1c levels were measured using high-performance liquid chromatography (Hemoglobin Analyzer D-10, Bio-Rad Laboratories, Berkeley, CA, USA). Levels of fasting serum-free triiodothyronine (FT3, reference interval: 3.5–6.5 pmol/L), serum-free thyroxine (FT4, reference interval: 11.5–22.7 pmol/L), serum thyroid-stimulating hormone (TSH, reference interval: 0.35–5.50 mIU/L), TGAb (reference interval: 0–110 IU/mL), TPOAb (reference interval: 0–40 IU/mL), and TRAb (reference interval: <10 U/L) were measured using an electrochemiluminescence analyzer (Cobas e601, Roche, Basel, Switzerland).

All subjects were advised to refrain from vigorous exercise before providing urine samples.24-hour urine samples were used to measure urine albumin levels using a chemiluminescence assay (Siemens Healthcare Diagnostics Products, L2KHA2). DN was defined as an albumin excretion rate (AER) higher than or equal to 30 mg/24 hours with at least two consecutive timed urine collections within a 3–6 month period [18]. According to the albumin excretion rate (AER), patients were divided into three categories: normoalbuminuria (< 30 mg/24 hours), microalbuminuria(≥ 30 but< 300 mg/24 hours), and macroalbuminuria(≥ 300 mg/24 hours).

### Statistical methods

All statistical analyses were carried out using SPSS 17.0 for Windows (Chicago, IL, USA). Variables with a normal distribution were expressed as the mean ± standard deviation (SD) and those with an abnormal distribution were expressed as the median (interquartile range). The categorical variables were expressed as proportions. Differences in clinical and laboratory values between patients with normoalbuminuria, microalbuminuria, and macroalbuminuria were assessed by a Pearson chi-square test, Wilcoxon test, or ANOVA. Serum levels of FT3, FT4, and TSH were assessed categorically (in tertiles) and continuously (per-SD change). Logistic regression analysis was used to assess the association between thyroid status and DN. We initially adjusted for age and sex (model 1) and in addition for smoking status, hyperlipidemia, the duration of diabetes, BMI, SBP, HbA1c level, and eGFR (model 2). Differences in DN prevalence by FT3, FT4, or TSH levels were calculated by the chi-square test. The statistical tests were two-sided, and a *P* value less than 0.05 was considered statistically significant.

## References

[1] Xu Y, Wang L, He J, Bi Y, Li M, Wang T, Wang L, Jiang Y, Dai M, Lu J, Xu M, Li Y, Hu N, 2010 China Noncommunicable Disease Surveillance Group (2013). Prevalence and control of diabetes in Chinese adults. JAMA.

[2] Menke A, Casagrande S, Geiss L, Cowie CC (2015). Prevalence of and Trends in Diabetes Among Adults in the United States, 1988-2012. JAMA.

[3] Tuttle KR, Bakris GL, Bilous RW, Chiang JL, de Boer IH, Goldstein-Fuchs J, Hirsch IB, Kalantar-Zadeh K, Narva AS, Navaneethan SD, Neumiller JJ, Patel UD, Ratner RE (2014). Diabetic kidney disease: a report from an ADA Consensus Conference. Diabetes Care.

[4] Dounousi E, Duni A, Leivaditis K, Vaios V, Eleftheriadis T, Liakopoulos V (2015). Improvements in the Management of Diabetic Nephropathy. Rev Diabet Stud.

[5] Iglesias P, Bajo MA, Selgas R, Diez JJ (2017). Thyroid dysfunction and kidney disease: An update. Rev Endocr Metab Disord.

[6] Iglesias P, Diez JJ (2009). Thyroid dysfunction and kidney disease. Eur J Endocrinol.

[7] Gopinath B, Harris DC, Wall JR, Kifley A, Mitchell P (2013). Relationship between thyroid dysfunction and chronic kidney disease in community-dwelling older adults. Maturitas.

[8] Woodward A, McCann S, Al-Jubouri M (2008). The relationship between estimated glomerular filtration rate and thyroid function: an observational study. Ann Clin Biochem.

[9] Jia F, Tian J, Deng F, Yang G, Long M, Cheng W, Wang B, Wu J, Liu D (2015). Subclinical hypothyroidism and the associations with macrovascular complications and chronic kidney disease in patients with Type 2 diabetes. Diabet Med.

[10] El-Eshmawy MM, Abd El-Hafez HA, El Shabrawy WO, Abdel Aal IA (2014). Response: subclinical hypothyroidism is independently associated with microalbuminuria in a cohort of prediabetic egyptian adults (diabetes metab j 2013;37: 450-7). Diabetes Metab J.

[11] Liu P, Liu R, Chen X, Chen Y, Wang D, Zhang F, Wang Y (2015). Can levothyroxine treatment reduce urinary albumin excretion rate in patients with early type 2 diabetic nephropathy and subclinical hypothyroidism? A randomized double-blind and placebo-controlled study. Curr Med Res Opin.

[12] Shin DH, Lee MJ, Lee HS, Oh HJ, Ko KI, Kim CH, Doh FM, Koo HM, Kim HR, Han JH, Park JT, Han SH, Yoo TH (2013). Thyroid hormone replacement therapy attenuates the decline of renal function in chronic kidney disease patients with subclinical hypothyroidism. Thyroid.

[13] Asvold BO, Bjoro T, Vatten LJ (2013). Associations of TSH levels within the reference range with future blood pressure and lipid concentrations: 11-year follow-up of the HUNT study. Eur J Endocrinol.

[14] Wang J, Zheng X, Sun M, Wang Z, Fu Q, Shi Y, Cao M, Zhu Z, Meng C, Mao J, Yang F, Huang X, Xu J (2015). Low serum free thyroxine concentrations associate with increased arterial stiffness in euthyroid subjects: a population-based cross-sectional study. Endocrine.

[15] Asvold BO, Bjoro T, Nilsen TI, Gunnell D, Vatten LJ (2008). Thyrotropin levels and risk of fatal coronary heart disease: the HUNT study. Arch Intern Med.

[16] Asvold BO, Bjoro T, Platou C, Vatten LJ (2012). Thyroid function and the risk of coronary heart disease: 12-year follow-up of the HUNT study in Norway. Clin Endocrinol (Oxf).

[17] Pearce SH, Brabant G, Duntas LH, Monzani F, Peeters RP, Razvi S, Wemeau JL (2013). ETA Guideline: Management of Subclinical Hypothyroidism. Eur Thyroid J.

[18] Fineberg D, Jandeleit-Dahm KA, Cooper ME (2013). Diabetic nephropathy: diagnosis and treatment. Nat Rev Endocrinol.

[19] Adler AI, Stratton IM, Neil HA, Yudkin JS, Matthews DR, Cull CA, Wright AD, Turner RC, Holman RR (2000). Association of systolic blood pressure with macrovascular and microvascular complications of type 2 diabetes (UKPDS 36): prospective observational study. BMJ.

[20] The Diabetes Control and Complications (DCCT) Research Group (1995). Effect of intensive therapy on the development and progression of diabetic nephropathy in the Diabetes Control and Complications Trial. Kidney Int.

[21] UK Prospective Diabetes Study (UKPDS) Group (1998). Intensive blood-glucose control with sulphonylureas or insulin compared with conventional treatment and risk of complications in patients with type 2 diabetes (UKPDS 33). Lancet.

[22] Pehlivan E, Ozen G, Taskapan H, Gunes G, Sahin I, Colak C (2016). Identifying the determinants of microalbuminuria in obese patients in primary care units: the effects of blood pressure, random plasma glucose and other risk factors. J Endocrinol Invest.

[23] Martin-Merino E, Fortuny J, Rivero-Ferrer E, Lind M, Garcia-Rodriguez LA (2016). Risk factors for diabetic retinopathy in people with Type 2 diabetes: A case-control study in a UK primary care setting. Prim Care Diabetes.

[24] Furukawa S, Yamamoto S, Todo Y, Maruyama K, Miyake T, Ueda T, Niiya T, Senba T, Torisu M, Kumagi T, Miyauchi S, Sakai T, Minami H (2014). Association between subclinical hypothyroidism and diabetic nephropathy in patients with type 2 diabetes mellitus. Endocr J.

[25] Chen HS, Wu TE, Jap TS, Lu RA, Wang ML, Chen RL, Lin HD (2007). Subclinical hypothyroidism is a risk factor for nephropathy and cardiovascular diseases in Type 2 diabetic patients. Diabet Med.

[26] Bando Y, Ushiogi Y, Okafuji K, Toya D, Tanaka N, Miura S (2002). Non-autoimmune primary hypothyroidism in diabetic and non-diabetic chronic renal dysfunction. Exp Clin Endocrinol Diabetes.

[27] Ortega E, Koska J, Pannacciulli N, Bunt JC, Krakoff J (2008). Free triiodothyronine plasma concentrations are positively associated with insulin secretion in euthyroid individuals. Eur J Endocrinol.

[28] Fernandez-Real JM, Lopez-Bermejo A, Castro A, Casamitjana R, Ricart W (2006). Thyroid function is intrinsically linked to insulin sensitivity and endothelium-dependent vasodilation in healthy euthyroid subjects. J Clin Endocrinol Metab.

[29] Bo S, Gentile L, Castiglione A, Prandi V, Canil S, Ghigo E, Ciccone G (2012). C-peptide and the risk for incident complications and mortality in type 2 diabetic patients: a retrospective cohort study after a 14-year follow-up. Eur J Endocrinol.

[30] Yilmaz MI, Sonmez A, Karaman M, Ay SA, Saglam M, Yaman H, Kilic S, Eyileten T, Caglar K, Oguz Y, Vural A, Yenicesu M, Zoccali C (2011). Low triiodothyronine alters flow-mediated vasodilatation in advanced nondiabetic kidney disease. Am J Nephrol.

[31] Taddei S, Caraccio N, Virdis A, Dardano A, Versari D, Ghiadoni L, Salvetti A, Ferrannini E, Monzani F (2003). Impaired endothelium-dependent vasodilatation in subclinical hypothyroidism: beneficial effect of levothyroxine therapy. J Clin Endocrinol Metab.

[32] Schalkwijk CG, Stehouwer CD (2005). Vascular complications in diabetes mellitus: the role of endothelial dysfunction. Clin Sci (Lond).

[33] Wu GH, Kong FZ, Cheng QZ, Luo WF, Du XD (2014). Low T3 syndrome predicts severe neurological deficits of cerebral infarction inpatients with large artery artherosclerosis in internal carotid artery system. Neuro Endocrinol Lett.

[34] Ozcan KS, Osmonov D, Toprak E, Gungor B, Tatlisu A, Ekmekci A, Kaya A, Tayyareci G, Erdinler I (2014). Sick euthyroid syndrome is associated with poor prognosis in patients with ST segment elevation myocardial infarction undergoing primary percutaneous intervention. Cardiol J.

[35] Zoccali C, Mallamaci F, Tripepi G, Cutrupi S, Pizzini P (2006). Low triiodothyronine and survival in end-stage renal disease. Kidney Int.

[36] Molitch ME, Steffes M, Sun W, Rutledge B, Cleary P, de Boer IH, Zinman B, Lachin J (2010). Development and progression of renal insufficiency with and without albuminuria in adults with type 1 diabetes in the diabetes control and complications trial and the epidemiology of diabetes interventions and complications study. Diabetes Care.

[37] Molitch ME, DeFronzo RA, Franz MJ, Keane WF, Mogensen CE, Parving HH, Steffes MW (2004). Nephropathy in diabetes. Diabetes Care.

[38] Perkovic V, Verdon C, Ninomiya T, Barzi F, Cass A, Patel A, Jardine M, Gallagher M, Turnbull F, Chalmers J, Craig J, Huxley R (2008). The relationship between proteinuria and coronary risk: a systematic review and meta-analysis. PLoS Med.

[39] Levey AS, Bosch JP, Lewis JB, Greene T, Rogers N, Roth D, Modification of Diet in Renal Disease Study Group (1999). A more accurate method to estimate glomerular filtration rate from serum creatinine: a new prediction equation. Ann Intern Med.

